# Bacteriophages playing nice: Lysogenic bacteriophage replication stable in the human gut microbiota

**DOI:** 10.1016/j.isci.2023.106007

**Published:** 2023-01-18

**Authors:** Steven G. Sutcliffe, Alejandro Reyes, Corinne F. Maurice

**Affiliations:** 1 McGill Centre for Microbiome Research, McGill University, Montreal, QC, Canada; 2 Department of Microbiology & Immunology, McGill University, Montreal, QC, Canada; 3 Max Planck Tandem Group in Computational Biology, Department of Biological Sciences, Universidad de los Andes, Bogotá 111711, Colombia; 4 The Edison Family Center for Genome Sciences and Systems Biology, Washington University School of Medicine, St Louis, MO, USA

**Keywords:** Virology, Microbiology, Microbiome

## Abstract

Bacteriophages, viruses specific to bacteria, coexist with their bacterial hosts with limited diversity fluctuations in the guts of healthy individuals where they replicate mostly via lysogenic replication. This favors ‘piggy-back-the-winner’ over ‘kill-the-winner’ dynamics which are driven by lytic bacteriophage replication. Revisiting the deep-viral sequencing data of a healthy individual sampled over 2.4 years, we explore how these dynamics occur. Prophages found in assembled bacterial metagenomes were also found extra-cellularly, as induced phage particles (iPPs), likely derived from prophage activation. These iPPs were diverse and continually present in low abundance, relative to the highly abundant but less diverse lytic phage population. The continuous detection of low levels of iPPs suggests that spontaneous induction regularly occurs in this healthy individual, possibly allowing prophages to maintain their ability to replicate and avoiding degradation and loss from the gut microbiota.

## Highlights

• Recent bioinformatics advances can help determine gut bacteria-phage interactions
• Spontaneous prophage induction results in a stable diverse subpopulation of phages
• Spontaneous prophage induction maintains ‘Piggyback-the-winner’ dynamics in the gut
• This could explain how free phages are maintained over time in healthy individuals

## Graphical abstract

**Figure:**
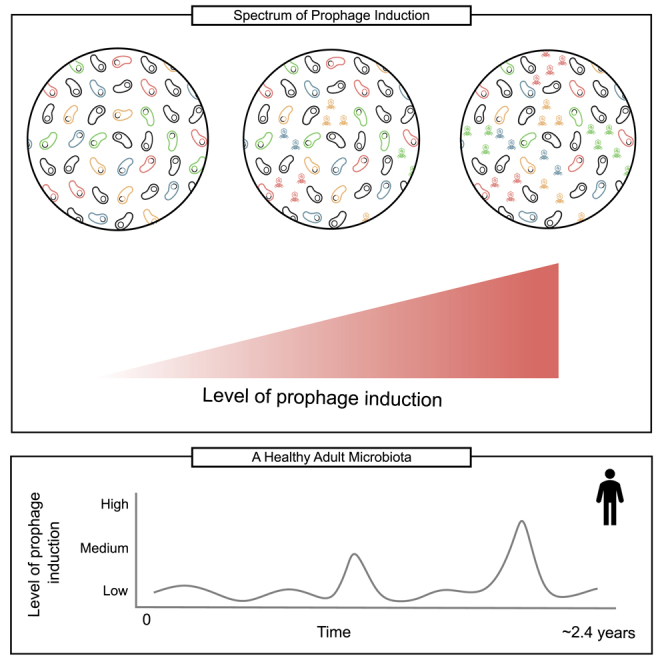

## Introduction

The human gut is home to a diverse and abundant community of microorganisms that are central to human health and development. The most abundant members of this community are bacteria, found in the trillions, and bacteriophages (abbreviated phages), with abundances in the same order of magnitude as bacteria.^1^ Phage and bacterial communities interact and co-evolve over the lifespan of the human host through a variety of infection dynamics^2^ shaped by their host’s age, diet, medication consumption, and disease.^2^^,^^3^^,^^4^ These tripartite host-bacteria-phage interactions result in an adult gut microbiota that is unique to each individual,^5^ with strongly correlated viral and bacterial communities.^6^ Bacteria-phage relationships are driven by complex dynamic interactions (see Abedon 2008^7^ for an overview), which range from strictly parasitic to symbiotic, depending on the phage lytic or lysogenic replication cycles, respectively.^2^^,^^8^ The lysogenic and lytic cycles differ by the integration of the phage genome as a prophage into the bacterial genome in the lysogenic cycle.^2^^,^^8^ Phages capable of lysogenic replication are referred to as temperate, and the bacterial host in which they integrate is a lysogen. Both lysogens^9^ and temperate phages^10^^,^^11^ have been observed in high abundance in the guts of healthy individuals, suggesting lysogeny is the dominant replication strategy in the gut. A longitudinal study of ten healthy adults further showed that the proportion of temperate phages is relatively consistent over time but their abundance differs between individuals.^5^ Understanding how lysogeny persists in the gut of healthy individuals will help contextualize the uniqueness of an individual’s gut virome,^5^ and the resulting viral-bacterial dynamics.^6^

Several types of bacteria-phage interactions and their downstream effects on microbial communities have been described to date.^2^^,^^12^^,^^13^ As one of the most extensively studied, ‘kill the winner’ dynamics refer to the Lotka-Volterra predator-prey dynamics existing between bacteria and phages replicating lytically, dependent on bacterial abundance and metabolism.^14^^,^^15^ In contrast, the increasing number of studies reporting dense populations of metabolically active bacteria with high rates of lysogeny, including in the gut, can be explained through ‘piggyback the winner’ dynamics.^16^^,^^17^ In this case, high bacterial density increases the rate of lysogeny by phage coinfection, or through host-density regulated molecular switches. Once integrated, prophages can provide a fitness advantage to their lysogen and protect them from further infection by superinfection exclusion or immunity, and the introduction of new genes encoding for virulence factors, antibiotic resistance, or novel metabolic functions. In this case, prophages persist by ‘making the winner’.^16^ Lysogeny in the adult healthy gut has been hypothesized to stem from ‘kill-the-winner’ dynamics that play out during infancy,^2^^,^^18^ leading to co-adaptation which stabilizes the interactions between phages and bacteria.^19^ The benefits of lysogeny for bacteria are counterbalanced by the competitive cost of prophages being additional genetic cargo that can switch to lytic replication through induction. Bacteria limit this switch by accumulating mutations within prophages at higher rates, rendering prophages inactive and incapable of lytic replication.^20^ Hence, it is important to distinguish active prophages, as those can still be induced, from inactive prophages, which are no longer capable of lytic replication. Prophage induction is typically triggered by extrinsic stimuli leading to DNA damage.^21^ In the mammalian gut, external factors such as diet^22^ and antibiotics^23^ have been shown to trigger prophage induction. Human pathologies, such as Crohn’s disease, could also lead to an increase in prophage induction.^24^ For their part, prophages in gut bacterial isolates have been induced by dietary compounds,^25^ short-chain-fatty acids,^26^ antibiotics, and other medications.^23^^,^^24^ Prophage induction can also occur in the absence of known external triggers, a process referred to as spontaneous induction,^27^ which may or may not be through the traditional bacterial SOS-response.^21^ Spontaneous prophage induction usually leads to a small subset of prophages undergoing lytic replication^28^ and is thought to be caused by intrinsic factors like stalled replication forks, stochastic gene expression or sporadic DNA damage,^21^ or could even result from intra-bacterial competition.^29^^,^^30^

Bacteria-phage interactions and dynamics are central to the resilience and maintenance of the gut microbiota. We therefore sought to determine how prophages could contribute to a healthy individual’s gut virome and hypothesized that most prophages in the gut can be induced and are therefore active prophages. In the absence of disease, antibiotic use, or drastic dietary changes, active prophages would translate to a small, yet stable fraction of extracellular induced phage particles (iPPs) present in the gut.

To test our hypothesis, we re-analyzed a previously published dataset of sequenced bacterial and viral metagenomic gut samples of a healthy individual over the course of 2.4 years.^31^ This dataset was selected based on its longitudinal sampling and sufficient resolution to identify prophages in bacterial metagenomes^32^ and detect induced phage particles (iPPs).^22^^,^^29^ With this *in silico* approach, we report that prophages contribute a continuous source of iPPs in this healthy individual most likely through spontaneous induction, despite also observing regular triggered prophage induction. Our results suggest evolutionary or adaptive constraints between bacteria and phages in the gut that limit highly disruptive widespread prophage induction events in favor of smaller, more targeted spontaneous prophage induction.

## Results

### Bacterial lysogens containing multiple active prophages are common in the gut of one healthy individual

**Figure 1:**
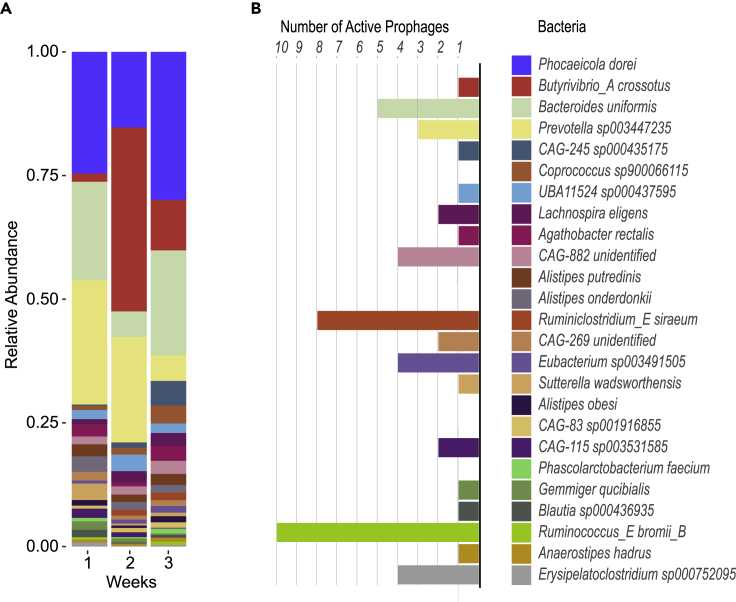
Distribution of bacterial lysogens in the gut of one healthy individual (A) Relative abundance of all (25) medium-to-high quality assembled bacterial bins from metagenomic sequencing for each experimental week over the 2.4 years. (B) Number of putative-active prophages in each bacterial bin. The color-coding of bacterial bins is the same between the two panels.

To study the prophages present within the genomes of bacterial lysogens, we used whole community metagenomic sequences and assembled 25 medium-to-high quality bacterial bins. All bacterial bins were taxonomically identified at the genus level, and 23 at the species level. The assembled bacterial community consisted mostly of Firmicutes and Bacteroidetes and one Proteobacteria, *Sutterella wadsworthensis*, a commonly found gut bacteria. These bins represent approximately 46%, 56%, 54% of metagenomic aligned reads on days 182, 852, 881, respectively. Bacteria not represented in the bins were likely at too low abundance for assembly and binning of adequate quality bins for prophage detection. Four of the bacteria represented between 67 and 79% of the mapped reads: *Prevotella sp003447235*, *Phocaeicola dorei*, *Bacteroides uniformis*, and *Butyrivibrio_A crossotus* (Figure 1A). Our bacterial diversity and bacterial bin abundances data are in line with what was reported in the original work using read-based methods.^31^

Each bacterial bin was investigated for prophages using multiple tools (see STAR Methods). Most prophages were detected by several tools (Figure S2A), which led to the identification of 651 non-redundant putative prophages (Figure S2B). We used Propagate^33^ to separate prophage-like or cryptic prophages from true prophages in the assembled bacterial bins, resulting in 52 putative active prophages (Figure S2C). We excluded the other predicted prophages from the analysis because it is impossible for us to differentiate if they are active but just not induced throughout the experiment, or are truly inactive (prophage-like sequences). We quantified that most bacteria (72%) contain at least one putative active prophage, and 40% of these bacterial lysogens contain multiple putative active prophages (Figure 1B).

### Active prophages are found extracellularly continuously at low levels as induced phage particles (iPPs)

**Figure 2:**
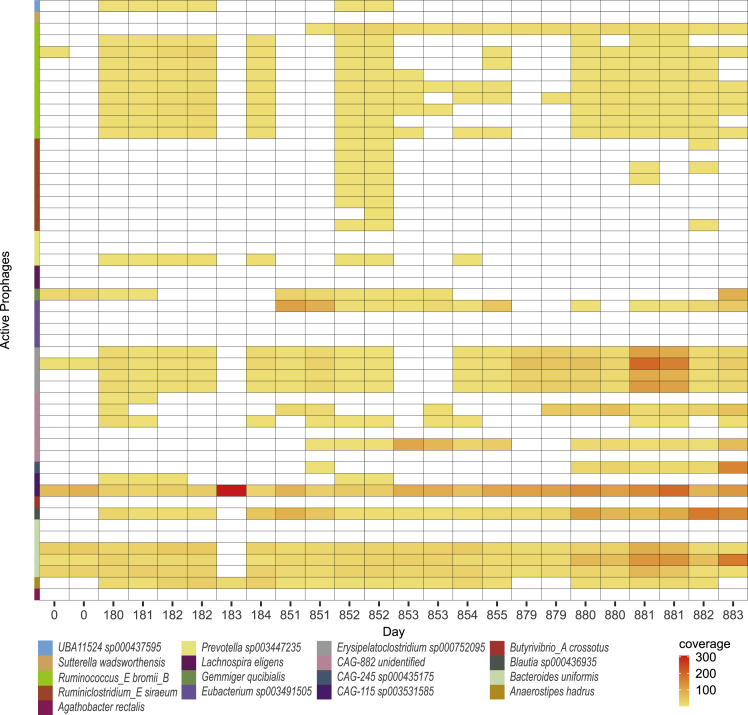
Presence of active prophages in the gut of one individual over 2.4 years Heatmap showing the relative abundance (coverage) of iPPs (rows) in each of the viral-enriched sampled time points. White time points indicate an absence of iPP in sample. Presence was defined as viral reads covering prophage regions by at least 1x for more than 75% of prophage length in the sample. A total of 38 out of 52 iPPs were considered present in at least one time point and determined active prophages. Normalized coverage of iPPs is displayed on timepoints when an active prophage was present in the sample.

**Figure 3:**
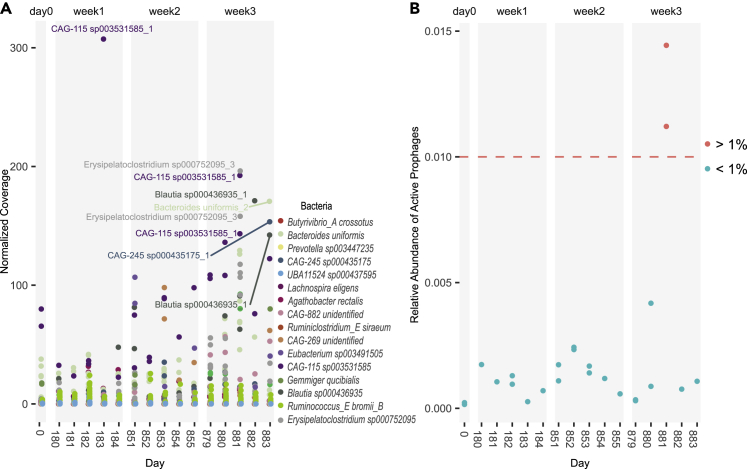
Spontaneous induction of prophages in a healthy individual dominates in comparison to triggered prophage induction events (A) Normalized coverage of viral reads aligned to each iPP over the course of the experiment. iPPs with significantly increased coverage (*Z* score>1.96) are labeled. Labels are color-coded according to the bacterial host. (B) Relative abundance of all viral reads aligning to iPP. Red time points are above the 1% relative abundance (dashed line).

We next aligned all the viral metagenomic reads to see when putative active prophages were found in the extracellular virome fraction as iPPs over the 2.4 years (Figure 2). Thirty-eight (73%) of the putative active prophages detected in the bacterial MAGs were confirmed as active prophages by iPP presence (Figure 2). In other words, 56% of our bacterial MAGs contain at least one confirmed active prophage. More than half of iPPs were found at 9 separate time points and three different weeks (Figure 2). Over the 2.4 years, 19 iPPs significantly increased in abundance during at least one time point compared to the other time points (DESEQ2, including zero-coverage timepoints, adjusted for multiple comparison, pvalue < 0.05). Eight iPPs found within six different bacterial lysogens reached DESEQ2 normalized coverage 100x (Figure 3A), and of those, five were significantly increased (*Z* score>1.96 of log-transformed coverage, including zero-coverage timepoints) at one time point. A significant increase in coverage fits with traditional models of triggered prophage induction. Significant abundance increase occurred almost entirely during week 3, between days 881–885 (Figure 3A). *CAG-115 sp003531585* prophage 1 was the exception, as it rose to significant abundance during week 1 (day 184) as well as week 3 (day 881) (Figure 3A). Significantly increased iPPs at week 3 were found to originate from five different host-bacteria belonging to both Firmicutes and Bacteroidetes; meaning the stimuli leading to the triggered induction of these active prophages was not phyla-specific. This contrasts with week 1 where triggered prophage induction was a species-specific event (Figure 3A). Despite coverage increasing significantly during week 1 and 3, iPPs represented at most 0.5% of the virome, with the exception of day 881, which rose above 1% (Figure 3B). The low relative abundance of iPPs (Figure 3B) and continuous detection over the 16 sampling times (Figure 2), together support a model of low, continuous spontaneous induction in the gut. This contrasts with the more typical triggered prophage induction, as seen on day 881, which leads to an increase in the relative abundance over 1% (Figure 3B).

### Prophage induction transiently alters extracellular phage communities

**Figure 4:**
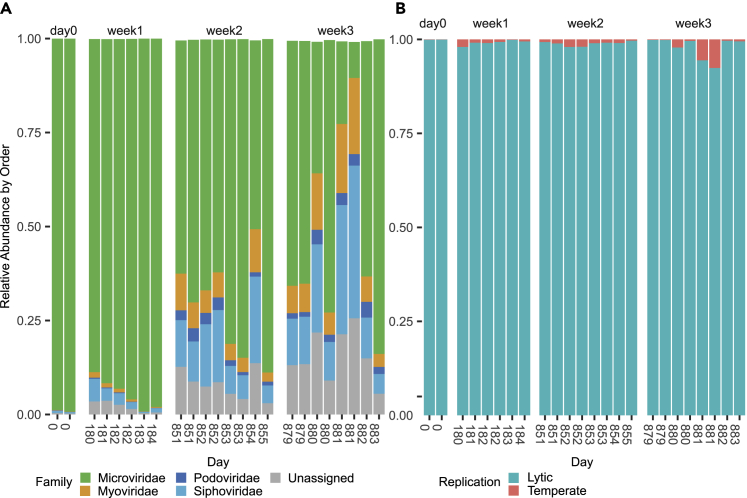
Diversity of phages in the gut of a healthy individual over 2.4 years (A) Relative abundance at the phage family-level showing disruption of stability at day 881 by triggered prophage induction. (B) Relative abundance of subset of high-quality viral contigs predicted to be either temperate or strictly lytic.

Having identified iPPs and their bacterial lysogens, we next set out to compare how spontaneous or triggered induction would alter the extracellular gut phage communities. From the 5,890 non-redundant viral contigs, we were able to classify 44% of these phage contigs taxonomically at the family-level (Figure S3). A large percentage of viral reads at weeks 2 and 3 were from unknown phages (Figure S3). The relative abundance of classified phages at the family-level shows most members belonging to the Microviridae family (Figure 4A), which contrasted with the absence of CrAss-like-phages. CrAss-like-phages have also been observed to be at low-abundance in individuals with a high-abundance of Microviridae phages.^5^ At the family-level, we see an expansion of families belonging to *Caudovirales* (Siphoviridae and Myoviridae) and unassigned phages at day 881 (Figure 4A). The expansion of Siphoviridae and Myoviridae in the virome at day 881 (Figure 4A) coincides with the increase in iPPs (Figure 3B). Triggered prophage induction, unlike spontaneous prophage induction, can rapidly alter the phage community diversity. Here, the effect of the prophage induction of day 881 appears transient, only lasting one day as Microviridae phages return to their high relative abundance the following day (day 882). In contrast, the triggered prophage induction observed on day 183, only impacted *CAG-115 sp003531585* and did not have an impact on viral diversity, likely because of induction only affecting one lysogen, unlike day 881, which affected five different lysogens.

To determine if iPPs can alter the proportion of extracellular temperate phages in the gut, we determined which of our phage contigs are potentially temperate using Bacphlip.^34^ Lysogenic replication cycle prediction by Bacphlip is designed to be run on complete phage genomes, as incomplete genomes underestimate temperate phages because of an absence of genetic hallmarks. For temperate phage analysis, we took a subset of our phage contigs that were high-quality (>90% complete) or complete by CheckV.^35^ This resulted in 557 phage contigs that represented a mean 82% (std. dev. 7.94) of viral reads. We found temperate phages represented a small relative abundance (Figure 4B). At week 3, we observed an increase in the temperate fraction of extracellular phages from an average 1.3%–2.1% and peaking on day 881 at 6.5% (Figure 4B). The increase was not significant but provides more evidence that the triggered prophage induction of day 881 does temporarily alter the phage community.

### iPPs are a stable yet divergent fraction of the gut phage communities

**Figure 5:**
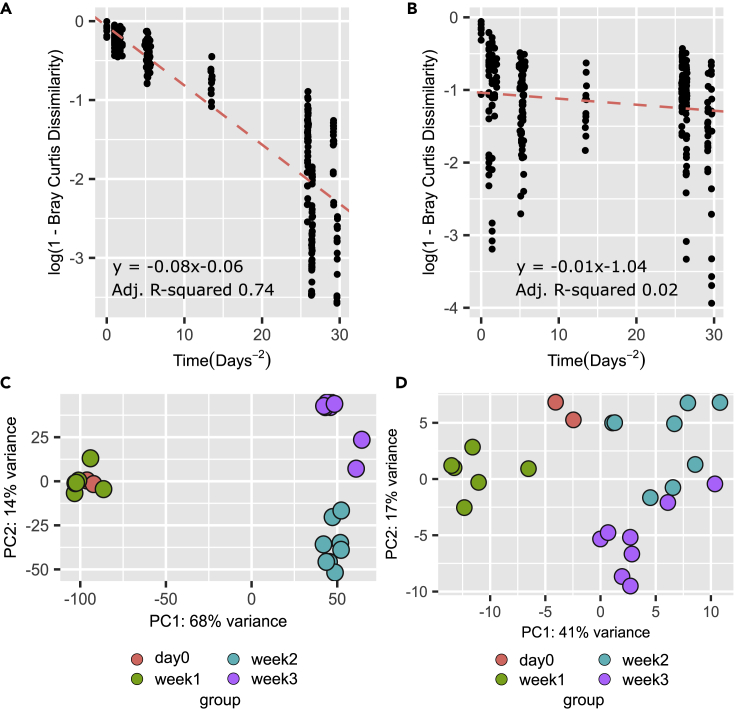
Comparison of the diversity and divergence of the gut virome to iPPs over 2.4 years (A–D) Log(1-Bray-Curtis Dissimilarity) was measured between all samples by time elapsed distance to track the degree the rate of community change (slope of dotted line) in both the (A) the whole gut virome negative slope (−0.08), and linear (adj. R-squared 0.74) of a community showing low initial diversity (intercept −0.06) and the (B) iPPs with a less negative slope, closer to zero (−0.01 adj. R-squared 0.02) but a high initial diversity (intercept −1.04) (C). PCA plot of DESEQ2 relative abundance of samples by time in the whole viral community (D) and in the iPP fraction.

To understand how the observed prophage induction dynamics shape the iPPs compared to the rest of the gut virome, we looked to discriminate between different patterns of community composition change (stochastic variation, directional change, and cyclical dynamics) using the approach of Collins et al.^36^ between the iPPs and the whole gut virome. We investigated the change in phage taxonomic composition with beta-diversity (log(1-Bray-Curtis dissimilarity)) between time points of the whole gut virome (5,890 phage contigs, including zero-abundant contigs) over time. The gut virome was initially very similar (intercept −0.06) but diverged significantly over time (represented by the negative sloped regression line −0.08 with multiple comparison adj. R-squared 0.7) indicating the community is diverging over time, becoming increasing dissimilar (Figure 5A). In comparison, the iPPs are initially more diverse (intercept −1.04) but undergo less divergence over time with a lower and non-significant negative slope (−0.01, adj. R-squared 0.002) (Figure 5B). iPPs thus differ from the gut virome as a whole, both being more diverse and showing less divergence. To confirm that the results for iPPs are not a sub-sampling artifact, we randomly sub-sampled 52 phage contigs from the whole community (20 iterations), and all confirmed the results. The rate of divergence over 2.4 years for the gut virome creates three compositionally distinct phage communities that significantly grouped together by week (PERMANOVA Pr(>F) 0.001: day 0/week 1 (days 0–184), week 2 (days 851–855), and week 3 (days 879–883), with later groups clustering closer because of temporal proximity (Figure 5C). The iPP population, initially more distant, with less temporal divergence, did not cluster by week (PERMANOVA Pr(>F) 0.11) (Figure 5D). The slower divergence rates of these diverse iPPs lead to less separation by week. The iPPs thus appear to be a diverse but stable community of extracellular phages maintained over long periods of time, despite prophage induction events.

We confirm that active prophages are commonly found in the genomes of gut bacteria, and that they replicate mainly through continuous spontaneous prophage induction. In addition, in the 16 time points sampled over 2.4 years, triggered prophage induction was detected four times, one of which involved multiple lysogens, disrupted viral composition at a family-level, and increased the abundance of extracellular temperate phages.

## Discussion

By revisiting bacterial and viral data from a healthy individual sampled over 2.4 years, we highlight that active prophages of lysogens in the gut are capable of induction and contribute to the extracellular virome, as induced phage particles (iPPs). Previous studies suggest that both lysogens^9^ and temperate phages^10^^,^^11^ are prevalent in the guts of healthy individuals, yet with high inter-individual variability in the relative abundance of temperate phages.^5^ How these are sustained in a highly productive system as the gut remains unclear. Here, our bioinformatics analyzes suggest that low abundances of temperate phages can be maintained through spontaneous induction of active prophages. Not all iPPs detected were the result of spontaneous prophage induction, as we also report two triggered prophage induction events. Those were restricted in time, transient, and limited to a few lysogens (5 out of 18). Numerous factors such as age, disease, drugs, and diet^3^^,^^4^ can result in prophage induction and alter both bacterial and viral compositions in the gut. It appears that in the absence of disease or antibiotics, this individual had two distinct triggered prophage induction events. The first event was targeted and species-specific, whereas the second one impacted multiple bacteria belonging to both Bacteroidetes and Firmicutes phyla, suggesting a more widespread environmental factor responsible. Unfortunately, no metadata was collected in the original work, so we hypothesize that non-antibiotic medication consumption^23^ or a switch in diet^22^ could be responsible. The *in silico* nature of the work detailed here does not allow for the unambiguous characterization of prophages and the newly produced iPPs, which has been extensively discussed elsewhere.^37^^,^^38^^,^^39^ There is also no information about the possible inducers and the mechanisms underlying prophage induction, whether spontaneous or triggered. Moving forward, these hypotheses need to be tested experimentally to better understand how species-specific *vs.* community-level triggers of prophage induction differ from each other. Large-scale studies with comprehensive metadata are needed^40^ or implementing gnotobiotic mouse models to explore features causing the induction of gut prophages *in vivo*.^29^ Importantly, such studies should incorporate longitudinal sampling, which is essential to unravel phage-bacteria interactions and detect prophage induction at the community level^22^^,^^31^^,^^41^^,^^42^^,^^43^

Prophages are common in the genomes of bacterial isolates,^44^ including in host-associated bacteria.^22^^,^^45^ It has been argued that prophages, especially active prophages, represent a fitness cost to their bacterial lysogen, as they represent extra genetic cargo that can act as a ‘molecular time-bomb’,^46^ and therefore, should be under selective pressure to be rendered inactive.^20^ Despite this, we report that most bacterial bins assembled contain active prophages. We propose that the low-level prophage induction observed, unrelated to bacterial abundance, allows for prophages to complete their infection cycle. This low-level prophage induction contrasts with the ‘molecular time-bomb’ model of prophage induction and instead could be a way for prophages to avoid the selective pressure of long-term integration and inactivation, similar to the toxin-antitoxin system.^10^ Lysogeny can thus be a mechanism for how temperate phages mitigate the fitness cost of actively replicating (reviewed in^21^). Bacteria have also been observed to benefit from this form of replication as the release of iPPs can act as ‘bacterial warfare’ to closely related bacteria vulnerable to infection, ultimately promoting lysogeny in the long term.^47^ Taken together, our results support the hypothesis that spontaneous prophage induction is a mechanism by which prophages maintain their ability to remain active over long periods of time.^21^^,^^48^

In contrast with the original study, we report that at the contig level, the gut virome of this individual is undergoing directional change. Indeed, phage contigs, which represent phage species or strains, were previously reported to be stable in this dataset, as 80% of them were found at the start and end of the study.^31^ The original findings focused on the most abundant phage contigs through manual curation and the Jaccard index, which focuses on the number of shared contigs. Since then, progress has been made to automate the identification of phage contigs using command-line tools (e.g., VirSorter^49^), annotated phage protein (e.g., pVOG^50^), and more extensive databases of gut viruses.^51^ These improvements allowed us to include rare phage contigs and use Bray Curtis dissimilarity, which includes contig relative abundance as well as presence/absence of contigs between time points, in our re-analysis. This allowed us to identify that iPPs undergo less compositional divergence over time than expected from the whole phage community, even with prophage induction. Our work supports the hypothesis that lysogeny limits phage genetic divergence in the gut.^19^ Prophages in the gut of this healthy individual appear to be balancing the benefits of stable integration with the risk of inactivation through regular low levels of spontaneous prophage induction. Increased triggered prophage induction would increase the fitness costs of harboring prophages and increase the selective pressures for prophage inactivation. In conclusion, bacteria and temperate phages balance competing priorities to form a stable equilibrium in the gut and play nice.

### Limitations of the study

This study is an *in silico* re-analysis of the landmark paper of Minot and colleagues,^31^ where we leveraged recent progress in bioinformatic tools to analyze phage genomes in complex communities. Thus, this work has inherent limitations associated with the current analytical tools available, namely bacterial genome assembly, the unambiguous identification of phages from phage-like elements, or the accurate detection of prophages. Nevertheless, our re-analysis puts forward some hypotheses about ‘piggyback-the-winner’ dynamics and the role of low spontaneous prophage induction and its effects on bacterial and phage communities. Experimental confirmation of these hypotheses will be key to better understand phage-bacteria interactions in communities where lysogeny is widespread.

## STAR★Methods

### Key resources table

**Table:** 

REAGENT or RESOURCE	SOURCE	IDENTIFIER
**Software and algorithms**		

Trimmomatic V.0.36	Bolger et al. 2014^52^	usadellab.org
Spades V.3.13.0	Prjibelski et al. 2020^53^^,^^54^	github.com/ablab/spades
CD-HIT-EST V4.8.1	Fu et al. 2012^55^^,^^56^	cd-hit.org/
VirSorter V.1.0.6	Roux et al. 2015^49^	github.com/simroux/VirSorter
bowtie2 V.2.3.5.1	Langmead et al. 2012^57^	bowtie-bio.sourceforge.net/bowtie2
megahit V.1.2.7	Li et al. 2016^58^	github.com/voutcn/megahit
MetaBat2 V.2.12.1	Kang et al. 2015^59^	bitbucket.org/berkeleylab/metabat
CONCOCT V.1.1.0	Alneberg et al. 2014^60^	github.com/BinPro/CONCOCT
MaxBin2 V. 2.2.7	Wu et al. 2016^61^	sourceforge.net/projects/maxbin2/
DAS-Tool V. 1.1.2	Sieber et al. 2018^62^	github.com/cmks/DAS_Tool
CheckM V.1.1.3	Parks et al. 2015^63^	ecogenomics.github.io/CheckM/
GTDB-Tk V1.4.1	Chaumeil et al. 2019^64^	github.com/Ecogenomics/GTDBTk
PHASTER	Arndt et al. 2016^66^	phaster.ca
VIBRANT V.1.2.1	Kieft et al. 2020^67^	github.com/AnantharamanLab/VIBRANT
PhageBoost V.0.1.7	Sirén et al. 2020^68^	github.com/ku-cbd/PhageBoost
mVIR V.1.0.0	Zund et al. 2021^69^	github.com/SushiLab/mVIRs
PropagAtE V.1.0.0	Kieft et al. 2022^33^	github.com/AnantharamanLab/PropagAtE
DESEQ2 V1.30.1	Love et al. 2014^70^	bioconductor.org/packages/release/bioc/html/DESeq2.html
VCONTACT2	Bin Jang et al. 2019^39^	bitbucket.org/MAVERICLab/vcontact2/wiki/Home
Demovir	NA	github.com/feargalr/Demovir
CheckV V.0.7.0	Nayfach et al. 2021^35^	bitbucket.org/berkeleylab/checkv/src/master/
Bacphlip V.0.9.6	Hockenberry et al. 2021^34^	github.com/adamhockenberry/bacphlip
R Stats V.4.2.1	R Foundation for Statistical Computing^72^	www.r-project.org/
Vegan V.2.6.2	Oksanen et al. 2013^73^	github.com/vegandevs/vegan
Ape V.5.6.2	Paradis et al. 2019^74^	ape-package.ird.fr/
ggplot2 V.3.3.6	Wickham et al. 2016^75^	ggplot2.tidyverse.org
UpSetR V.1.4.0	Conway et al. 2017^76^	gehlenborglab.org/research/projects/upsetr/

**Other**		

Scripts and Data for analysis	This paper	github.com/sgsutcliffe/Bacteriophages_Playing_Nice.git
Gut Virome Database	Gregory et al. 2020^51^	https://doi.org/10.25739/12sq-k039
crass-like-phage genomes	Guerin et al. 2020^71^	https://doi.org/10.1016/j.chom.2018.10.002
PVOG database	Grazziotin et al. 2017^50^	dmk-brain.ecn.uiowa.edu/pVOGs

### Resource availability

#### Lead contact

Further information and requests for resources or code should be directed to and will be fulfilled by the lead contacts Corinne Maurice (corinne.maurice@mcgill.ca) and Alejandro Reyes (a.reyes@uniandes.edu.co).

#### Materials availability

This study did not generate new unique reagents.

### Experimental model and subject details

#### Dataset

We used the previously published data of a healthy male generated by Minot and team, whose fecal samples were collected at sixteen time points spread over 884 days (_∼_2.4 years).^31^ Briefly, fecal samples were collected on four separate occasions: one sample was collected on the first day of the first week of the study, while one sample per day was collected for the three subsequent weeks (Figure S1A). The healthy twenty-three-year-old did not take antibiotics over the course of the experiment. The viral fraction was separated and sequenced at all sixteen timepoints, with eight timepoints sequenced twice (Figure S1A). Bacterial metagenomics were also obtained from the same fecal samples at three time points during the three last sample collection weeks (Figure S1A).^31^

### Method details

#### Viral assembly

An overview of the analytical pipeline for viral assembly is provided in Figure S1B. Sequence reads from viral-enriched libraries (See Minot et al. 2013 for details)^31^ were trimmed with Trimmomatic V.0.36,^52^ minimum quality 35 and minimum length of 70 (SLIDINGWINDOW:4:35 MINLEN:70 HEADCROP:10). As recommended,^38^ we assembled viral contigs for each sequence run separately with Spades V.3.13.0^53^ using the metaSpades option.^54^ Viral assembled contigs were pooled, resulting in 291,758 contigs. Contigs less than 1kb in length were removed, resulting in 24,845 viral contigs. We used CD-HIT-EST V4.8.1^55^^,^^56^ with 0.95 similarity threshold, 8-word size, 0.9 length cut-off to cluster the contigs from the different samples, resulting in 22,091 non-redundant viral contigs. We then selected for phage contigs, as those fulfilling at least one of the following three criteria: 1) Detected as viral by VirSorter (V.1.0.6) with custom database option additionally using the Gut Virome Database^51^; 2) at least three ORFs (predicted by Prodigal V.2.6.3 with metagenomic mode) with homology (HMMER V.3.1b2 hmmscan minimum e-value 1e-5) to PVOG database (Downloaded on Dec 1, 2020); or 3) BLASTn homology (e-value 1e-10, with 80% coverage of shortest contig) to Gut Virome Database.^51^ This resulted in 14,444 phage contigs, of which 6,176 viral contigs were greater than 2.5kb in length (Figure S1B).

#### Bacterial assembled genomes

An overview of the analytical pipeline for bacterial assembly is provided in Figure S1B. Bacterial metagenomic reads were trimmed with Trimmomatic V.0.36^52^ (LEADING:3 TRAILING:3 SLIDINGWINDOW:4:15 MINLEN:36) and decontaminated for human sequences by aligning reads to *Homo sapiens* GRCh38 genome with bowtie2 V.2.3.5.1.^57^ Remaining trimmed and decontaminated reads were pooled and assembled into contigs with megahit V.1.2.7^58^ using the default settings. We generated bacterial bins with contigs using MetaBat2 V.2.12.1^59^-m 1500 (41 bins), CONCOCT V.1.1.0^60^ (77 bins) and MaxBin2 V. 2.2.7^61^ (14 bins). Bins were merged using DAS-Tool V. 1.1.2.^62^ We used a score threshold of 0.35 to retrieve 27 bins. We then used CheckM V.1.1.3^63^ to confirm that all bins were unique. We selected bins that met the criteria of being either >40% complete and <10% contaminated by CheckM or having a DAS-Tool bin score of >0.4. This resulted in 25 medium-to-high quality bacterial bins (Figure S1B). We assigned taxonomy to the bins using GTDB-Tk V1.4.1^64^ using the reference database^65^ version r95. We determined the relative abundance as the percentage of reads that aligned to one of the 25 bins we detected. The total number of aligned reads per bin was normalized by bin size (Figure 1A).

#### Prophage detection and identifying active prophages

An overview of the analytical pipeline for the detection of phage contigs and active prophages is provided in Figure S1B. Prophages were detected within bacterial bins by combining various tools: PHASTER,^66^ VIRSorter (V.1.0.6),^49^ VIBRANT (V.1.2.1),^67^ PhageBoost (V.0.1.7),^68^ and mVIR (V.1.0.0).^69^ We also used a custom alignment method, where we aligned the viral reads to each bacterial bin using bowtie2 (V.2.3.5.1), then used samtools mpileup to calculate coverage per base (with default perimeters). Using a sliding window of 1,000bp over the entire genome, if the average coverage was >10x, we considered the region as a putative prophage region. In total, we found 2,719 putative prophages. We then merged putative prophage regions detected by multiple tools that overlapped, resulting in 1,844 putative prophages (Figure S2A). Out of these 1,844 putative prophages, 651 putative prophages met one of our three phage criteria (see viral assembly section). To distinguish between cryptic or defective prophages from prophages still capable of induction (active prophages), we then ran PropagAtE (v1.0.0),^33^ which detects prophages with coverage significantly higher than what would be expected based on bacterial genome coverage, on these remaining 651 putative prophages (default Cohen’s *d* test and prophage:host ratio). As not all of the detected prophages met the requirement of having host-bacterial flanking regions identified, we ran PropagAte with a modified script (available upon request) that replaced host-bacterial flanking region coverage with the entire bin coverage when the flanking host region of the prophage was less than 5bp in length. The risk of prophages being incorrectly binned increases in the absence of host-flanking regions^37^; however, this risk is mitigated in this study as bacterial bins with prophages were distantly related (> than genus level), which limits incorrect binning (Figure 1B). In addition, not having flanking regions we include potential phages undergoing chronic infection. Finally, we obtained 52 prophages predicted as active prophages, capable of being induced, and thereby contributing to the extracellular fraction of the virome as iPPs.

#### Viral community

The viral population used in this study consisted of 6,176 assembled phage contigs and 52 active prophages detected in bacterial MAGs. Out of the 6,176 assembled phage contigs, we removed 338 that had homology to the set of active prophages (BLASTn e-value 1e-5), resulting in a total of 5,890 non-redundant viral contigs. Quality trimmed reads were aligned to viral contigs using bowtie (V.2.3.5.1). Read coverage was normalized by sample using DESEQ2 V1.30.1^70^ and by viral contig length. Viral contigs were considered ‘present’ in a sample if their genome was covered in 75% of the length by at least 1x coverage.^38^ Family-level taxonomic classification was performed by using a voting-approach after comparing genes on the amino-acid level against the viral subset of TrEMBL by Demovir (github.com/feargalr/Demovir). No CrAss-like-phages were predicted, and their absence was confirmed through additional comparisons of our viral contigs against Guerin crAss-like phage genomes,^71^ through BLASTn similarity and shared viral clusters (using VCONTACT2^39^). Less than 0.05% of viral reads aligned to the Guerin crAss-like phage genomes,^71^ indicating a low-abundance of reads with homology to CrAss-like-phages in this individual.

Before predicting the replication strategy of viral contigs we selected the high-quality viral contigs (i.e., ≥40% complete and classified as high-quality by CheckV (V.0.7.0.^35^). These high-quality viral contigs (557) represent a mean of 82% (std. dev. 7.94) of quality controlled viral reads. We used Bacphlip V.0.9.6^34^ on our high-quality viral contigs to predict which were temperate (that is with a >50% chance of being temperate). Bacphlip is designed to be used on complete genomes and will under-report temperate phages when applied to incomplete or fragmented phage genomes.

### Quantification and statistical analysis

All statistical tests were run with R stats,^72^ except for beta-diversity, PERMANOVA (Vegan V.2.6.2^73^), normalization (DESEQ2 V.1.36.0^70^), and principal coordinate analysis (Ape V.5.6.2^74^) with p < 0.05 considered to be significant. Statistical analysis details can be found in the script https://github.com/sgsutcliffe/Bacteriophages_Playing_Nice/blob/main/VLP_analysis.R. Data was visualized using ggplot2 V.3.3.6^75^ with exception of Figure S2, which was generated using UpSetR V.1.4.0.^76^

## Data Availability

• This paper analyzes existing, publicly available data. These accession numbers for the datasets are listed in the key resources table. • All original code has been deposited at https://github.com/sgsutcliffe/Bacteriophages_Playing_Nice and is publicly available as of the date of publication. DOIs are listed in the key resources table. • Any additional information required to reanalyze the data reported in the paper is available from the lead contacts upon request.
