# Supplementary material for: Bacteriophages playing nice: Lysogenic bacteriophage replication stable in the human gut microbiota
Source: iScience. 2023 Jan 18;26(2):106007. doi: 10.1016/j.isci.2023.106007 (PMC9926308; doi:10.1016/j.isci.2023.106007)
Supplement: Document S1. Figures S1–S3 [file mmc1.pdf]

iScience, Volume *26*

Supplemental information

Bacteriophages playing nice: Lysogenic bacteriophage replication stable in the human gut microbiota

Steven G. Sutcliffe, Alejandro Reyes, and Corinne F. Maurice

Supplementary Material

Figure S1. Study Summary, Related to Methods and all figures.

(A) Explanation of the sampling for the study (B) Methodology for bacterial and viral analysis

Figure S2. Upset Plot Showing Overlap Between Prophage Predictors, Related to Figure 1.

Summary of prophage predictions by Bowtie, mvir, phageboost, vibrant, phaster, virsorter of (A) All the 2, 719 merged prophage regions (B) All the 651 prophages that meet our phage criteria (C) and the 52 active prophages

Figure S3. Percentage of Phages with Taxonomic Classification at the Family-Level per Sample-Sequence Run, Related to Figure 4.

Percentage of phages with taxonomic classification at the family-level per sample-sequence run. Individual contigs are separated by grey-lines showing the breakdown of individual contigs.

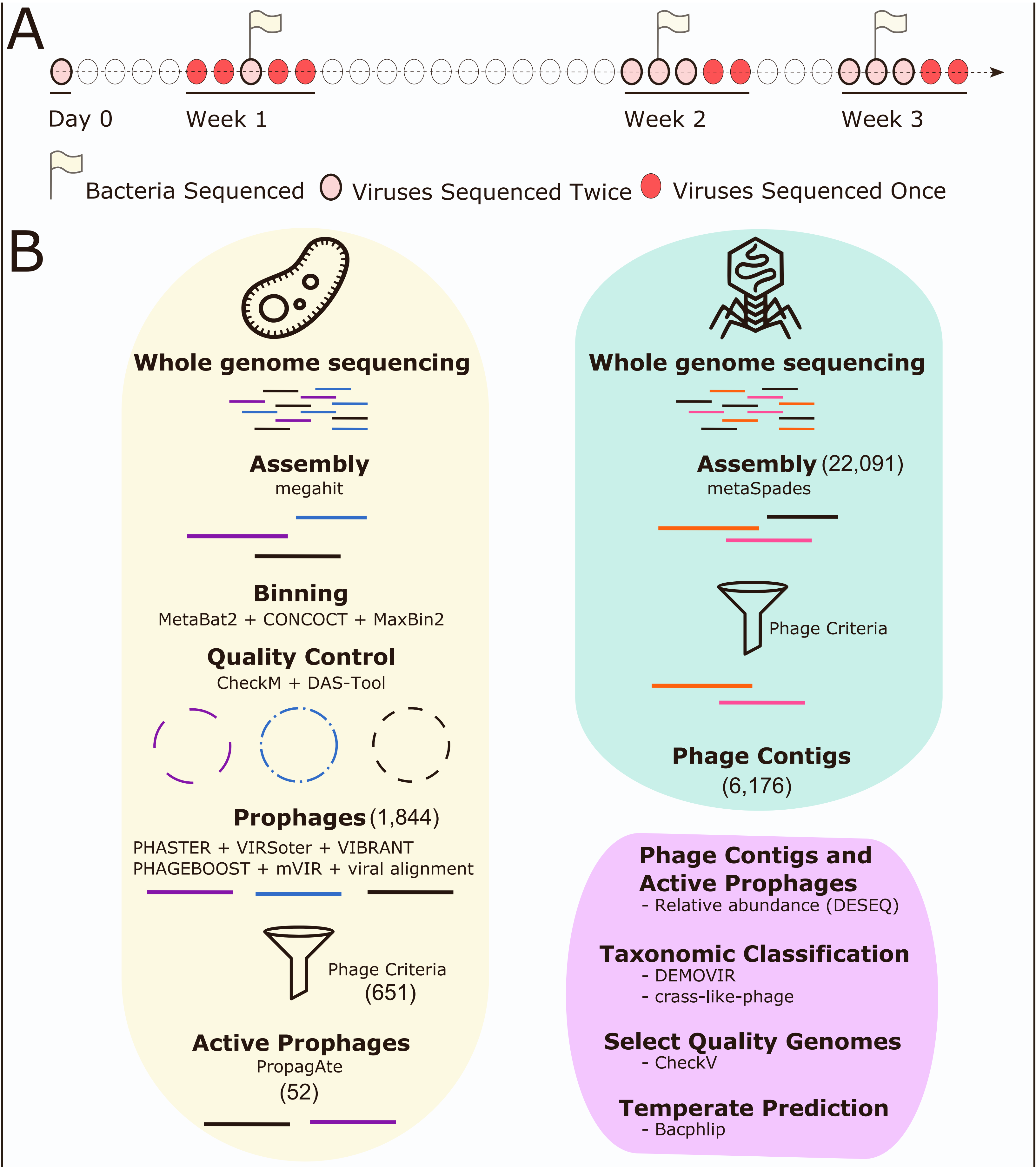
A
Day 0
Week 1
Week 2
Week 3
Bacteria Sequenced
Viruses Sequenced Twice
Viruses Sequenced Once
B
Whole genome sequencing
Assembly
megahit
Binning
MetaBat2 + CONCOCT + MaxBin2
Quality Control
CheckM + DAS-Tool
Prophages (1,844)
PHASTER + VIRSoter + VIBRANT
PHAGEBOOST + mVIR + viral alignment
Phage Criteria
(651)
Active Prophages
PropagAte
(52)
Whole genome sequencing
Assembly (22,091)
metaSpades
Phage Criteria
Phage Contigs
(6,176)
Phage Contigs and
Active Prophages
- Relative abundance (DESEQ)
Taxonomic Classification
- DEMOVIR
- crass-like-phage
Select Quality Genomes
- CheckV
Temperate Prediction
- Bacphlip

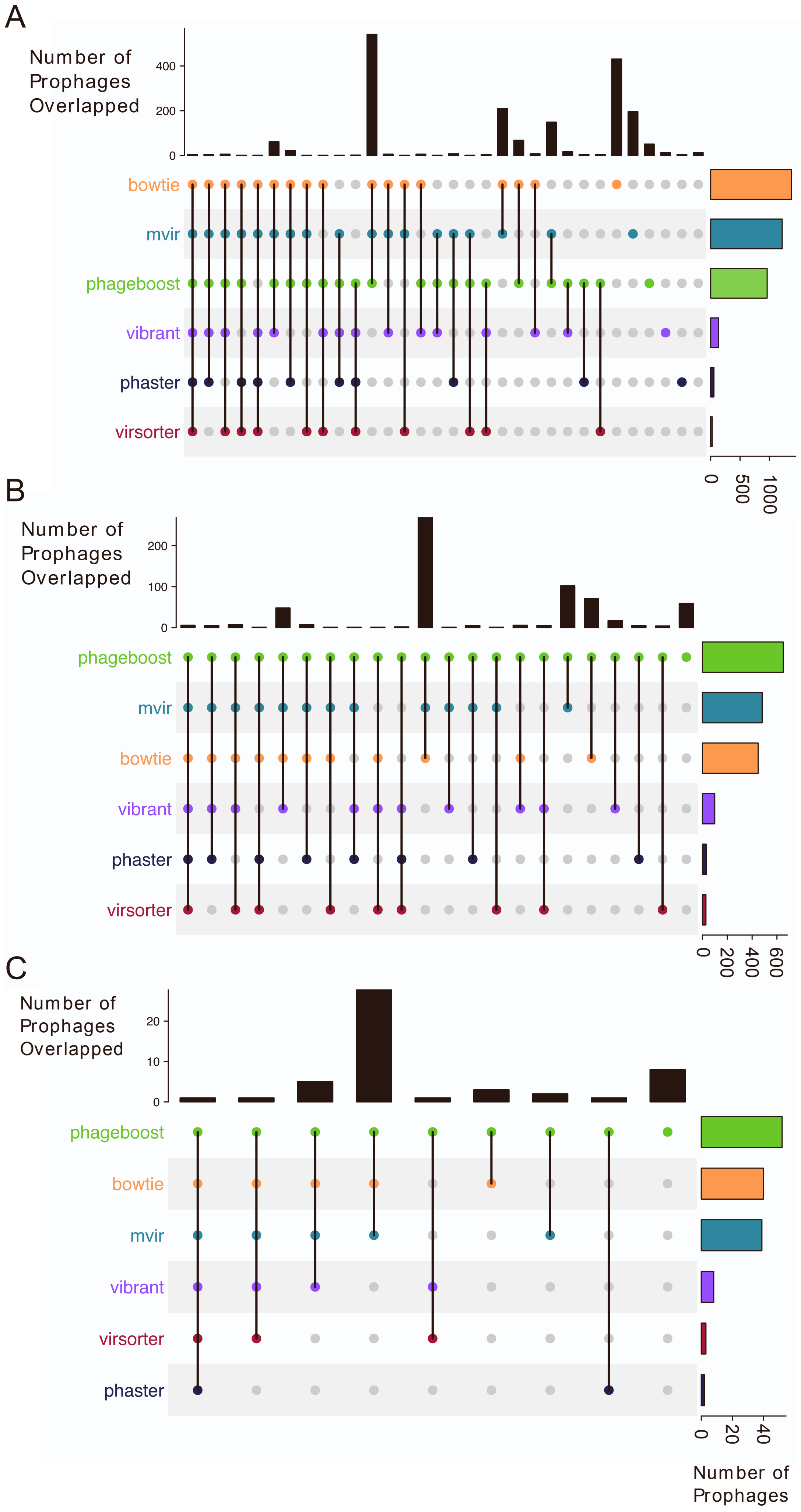

A
Number of Prophages Overlapped
400
200
0
bowtie
mvir
phageboost
vibrant
phaster
virsorter
0
500
1000
B
Number of Prophages Overlapped
200
100
0
phageboost
mvir
bowtie
vibrant
phaster
virsorter
0
200
400
600
C
Number of Prophages Overlapped
20
10
0
phageboost
bowtie
mvir
vibrant
virsorter
phaster
0
20
40
Number of Prophages

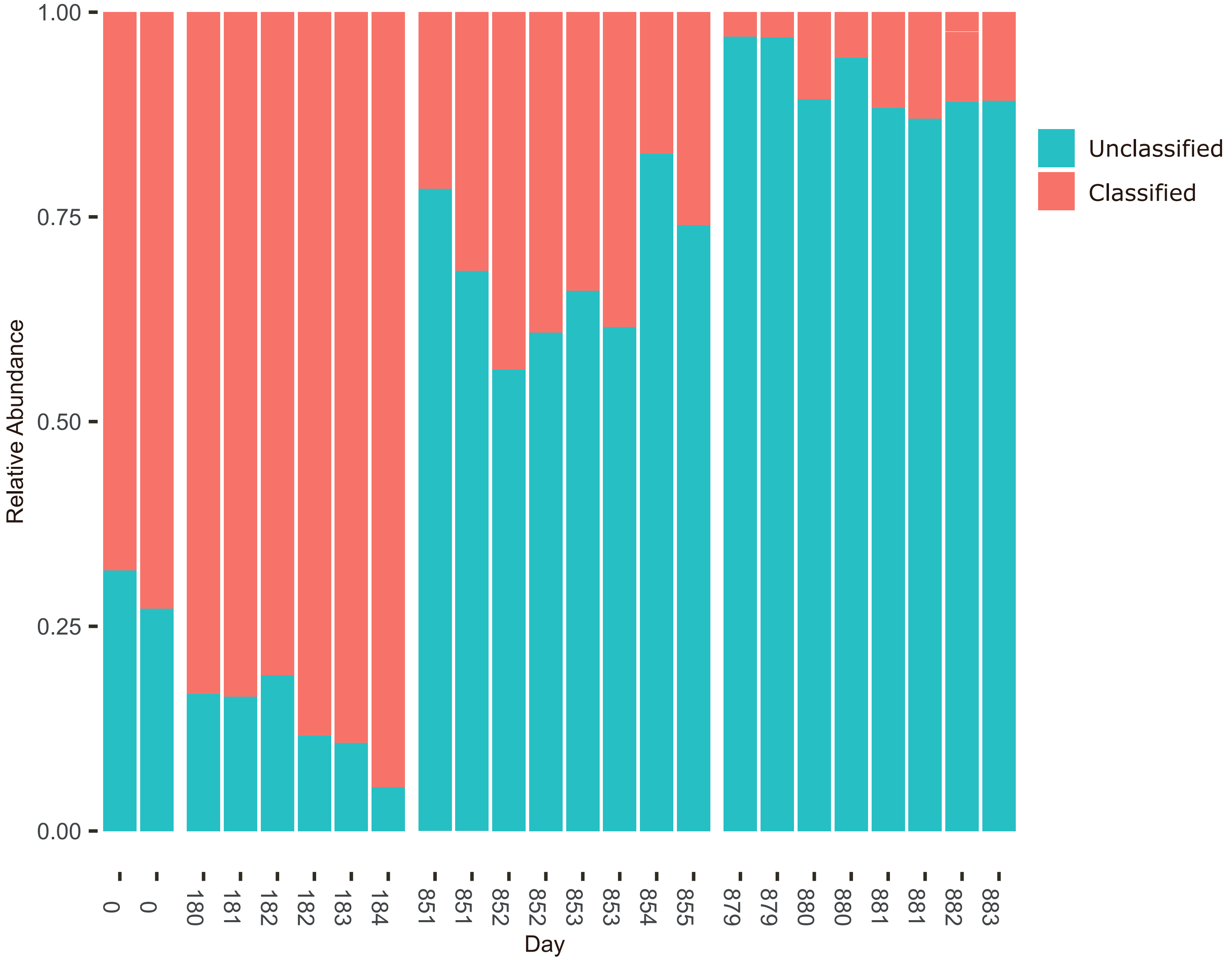

1.00
0.75
0.50
0.25
0.00
Relative Abundance
0
0
180
181
182
182
183
184
851
851
852
852
853
853
854
855
879
879
880
880
881
881
882
883
Day
Unclassified
Classified
